# Mechanisms of Risk Reduction in the Clinical Practice of Alzheimer’s Disease Prevention

**DOI:** 10.3389/fnagi.2018.00096

**Published:** 2018-04-10

**Authors:** Matthew W. Schelke, Peter Attia, Daniel J. Palenchar, Bob Kaplan, Monica Mureb, Christine A. Ganzer, Olivia Scheyer, Aneela Rahman, Robert Kachko, Robert Krikorian, Lisa Mosconi, Richard S. Isaacson

**Affiliations:** ^1^Vagelos College of Physicians and Surgeons, Columbia University, New York, NY, United States; ^2^Attia Medical, PC, San Diego, CA, United States; ^3^Weill Cornell Medicine, Cornell University, New York, NY, United States; ^4^Hunter College, City University of New York, New York, NY, United States; ^5^Inner Source Health, New York, NY, United States; ^6^College of Medicine, University of Cincinnati, Cincinnati, OH, United States

**Keywords:** Alzheimer’s disease, Alzheimer’s prevention, clinical precision medicine, precision medicine, glucose hypometabolism, inflammation, oxidative stress, calcium regulation

## Abstract

Alzheimer’s disease (AD) is a neurodegenerative dementia that affects nearly 50 million people worldwide and is a major source of morbidity, mortality, and healthcare expenditure. While there have been many attempts to develop disease-modifying therapies for late-onset AD, none have so far shown efficacy in humans. However, the long latency between the initial neuronal changes and onset of symptoms, the ability to identify patients at risk based on family history and genetic markers, and the emergence of AD biomarkers for preclinical disease suggests that early risk-reducing interventions may be able to decrease the incidence of, delay or prevent AD. In this review, we discuss six mechanisms—dysregulation of glucose metabolism, inflammation, oxidative stress, trophic factor release, amyloid burden, and calcium toxicity—involved in AD pathogenesis that offer promising targets for risk-reducing interventions. In addition, we offer a blueprint for a multi-modality AD risk reduction program that can be clinically implemented with the current state of knowledge. Focused risk reduction aimed at particular pathological factors may transform AD to a preventable disorder in select cases.

## Introduction

Alzheimer’s disease (AD) is a major source of morbidity and mortality worldwide, affecting one in eight individuals and costing over $400 billion annually (Wimo et al., 2013). The pathology of AD almost always involves accumulations of the proteins beta-amyloid (Aβ) in extracellular plaques and tau in intracellular neurofibrillary tangles, which likely represent the final stages of neuronal death (Hardy and Selkoe, 2002). Aβ, in particular, has been thought to play an important role in the pathogenesis of the disease by leading to inflammation, oxidative damage, and metabolic failure with subsequent widespread neuronal death and cerebral atrophy in advanced disease (Mattson, 2004).

Over the past three decades, many potential disease-modifying therapies directed at amyloid have been developed in model systems but have failed to demonstrate efficacy in Phase 3 human trials. A 2014 study identified 244 compounds tested in 413 clinical trials between 2002 and 2012, and found that only one (memantine) was approved for use by the United States Food and Drug Administration (FDA) (Lleo et al., 2006). Thus, the failure rate was 99.6%. The only available pharmacotherapies for AD are acetylcholinesterase inhibitors and memantine, both of which may improve clinical symptoms but do not modify the course of the disease (Lleo et al., 2006). These failures may be due to a number of plausible reasons. For example, the potential disease-modifying agents may have been administered too late in the disease process (Cummings et al., 2014); targeting amyloid alone is not sufficient to modify disease (i.e., other pathogenic mechanisms must also be targeted); or amyloid is a sequelae, rather than a cause, of AD pathology. If either of the latter two is correct, then it may be possible to reduce risk, delay or prevent AD through early modulation of pathologic pathways that precede clinically significant amyloid and tau accumulation.

From both a clinical and translational perspective, evidence suggests that AD risk reduction is feasible for several reasons (Galvin, 2017). First, AD risk has increased out of proportion to longevity, suggesting that there are factors other than age and genetic predisposition (i.e., environmental triggers) that contribute to the disease and may be modulated early in its course (Galvin, 2017). Second, there is a decades-long latency period from initial pathologic changes to the first symptoms, offering a wide window for early intervention (Morris, 2005). To further pursue the clinical significance of early intervention, several clinics were founded that are dedicated to AD risk assessment and early intervention, providing direct clinical care to at-risk patients (Isaacson, 2017). These programs provide targeted therapies for each patient by addressing multiple pathophysiologic mechanisms of AD (Hardy, 2009) that are not solely amyloid-based, such as poor cerebral perfusion from vascular disease (de la Torre, 2004) and cerebral glucose hypometabolism (Mosconi et al., 2008). As these pathways are influenced by many aspects of cerebral biology—glucose metabolism, inflammation, oxidative stress, trophic factor signaling, and calcium flux (Choi, 1994) – multi-modality AD risk-reducing strategies that collectively affect all or most of these mechanisms may be more effective than single-target therapy (i.e., amyloid reduction alone). The most accurate term to describe this approach is clinical precision medicine. An expanded clinical history (e.g., past and current lifestyle patterns, environmental exposures, life-course events) and physical/neurological examination are interpreted in conjunction with anthropometrics, blood biomarkers (including genetics), and cognitive performance (Seifan and Isaacson, 2015; Schelke et al., 2016). A comprehensive, multi-modal management plan is then carefully crafted targeting as many relevant factors as possible, rather than a “one-size-fits-all” approach.

**FIGURE 1:**
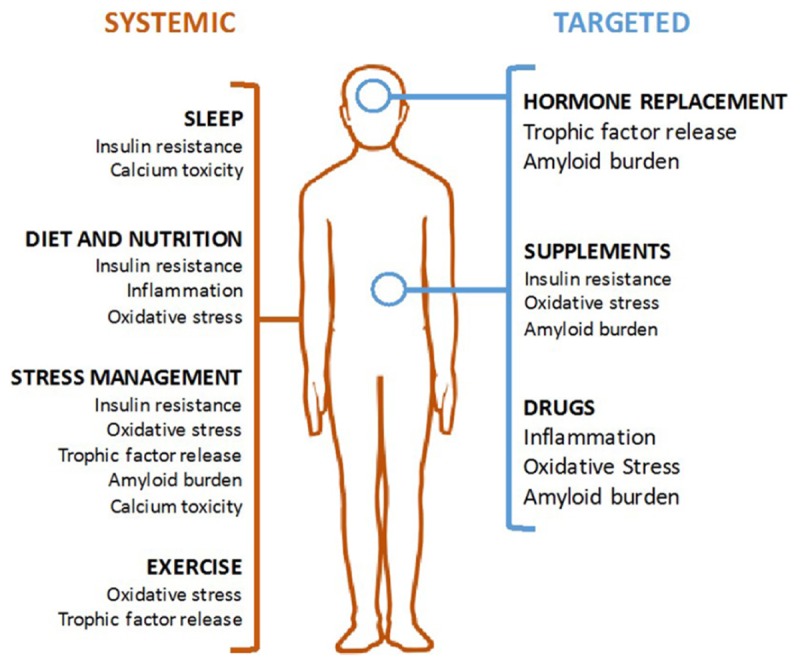
Modalities of Alzheimer’s disease (AD) prevention. Systemic interventions (in orange, on left) should be the foundation of any AD prevention program and are aimed at correcting an unhealthy baseline. Targeted interventions (in blue, on right) can be used for patient with specific indications.

This review outlines six mechanisms involved in AD pathogenesis and the corresponding interventions to modulate each mechanism and reduce AD risk (**Table 1**). To address the brain and body interplay of AD, these mechanisms can fall into two categories: those affecting systemic physiology (impaired glucose metabolism, inflammation and oxidative stress) and those more specific to neurologic mechanisms (trophic factors, amyloid burden, and calcium toxicity) (**Figure 1**). Finally, we offer a blueprint of clinical applications utilizing these risk-reducing interventions.

**Table 1:** Interventions for systemic and neurologic mechanisms in Alzheimer’s disease.

Category	Mechanism	Interventions
Systemic	Glucose metabolism and insulin resistance	Exercise Nutrition Sleep Stress management Supplements (e.g., alpha-lipoic acid)
	Inflammation	Drugs (e.g., NSAIDs) Nutrition Sleep Socialization
	Oxidative stress	Drugs (e.g., NSAIDs) Exercise Nutrition Stress management Supplements (e.g., B vitamins, PUFAs)
Neurologic	Trophic factors	Exercise Hormone replacement (E+P and T) Stress management
	Amyloid burden	Drugs (e.g., Aducanumab) Hormone replacement (E) Stress management
	Calcium toxicity	Supplements (e.g., Vitamin D) Sleep

## Mechanisms of Alzheimer’s Disease Pathogenesis

### Systemic Mechanisms of Alzheimer’s Disease Pathogenesis: Impaired Glucose Metabolism, Inflammation, and Oxidative Stress

#### Impaired Glucose Metabolism

Obesity, type 2 diabetes mellitus (T2D), and metabolic syndrome have been identified in epidemiological studies as major risk factors for AD (Blass et al., 2000; Ahtiluoto et al., 2010). The brains of AD patients demonstrate decreased expression of insulin and insulin-like growth factor (IGF) signaling components, consistent with neuronal insulin resistance early in the disease (Rivera et al., 2005), and correlated with worsening of AD progression over time. Additionally, cerebral glucose metabolism is reduced in patients with early amnesic mild cognitive impairment (MCI), which precedes AD dementia (Mosconi et al., 2008). Altogether, this evidence suggests that insulin resistance contributes to the pathogenesis of AD.

There is evidence that obesity and diabetes contribute to neuronal insulin resistance and AD pathology. Obesity is characterized by excess body fat, adipose tissue dysregulation, chronic elevations of inflammatory cytokines, including tumor necrosis factor-alpha (TNF-a), leptin, interleukin 1 beta (IL-1B), and interleukin 6 (IL-6), and increased c-Jun N-terminal kinase (JNK) signaling, which often coincides with T2D (Hirosumi et al., 2002). Insulin resistance at the muscle and adipose tissue, the hallmark of T2D, results in hyperglycemia and compensatory hyperinsulinemia. Hyperinsulinemia, which often results from early systemic insulin resistance, also promotes neuroinflammation and Aβ deposition (Takeda et al., 2010). Elevations in glucose levels have been shown to prospectively relate to an increased risk of dementia. This dose-dependent relationship occurs in persons both with and without diabetes (Crane et al., 2013).

Peripheral insulin resistance may contribute to neuronal insulin resistance through multiple mechanisms. In Tg2576 mice, a high-fat-high-sugar diet induced insulin resistance, decreased central insulin receptor-mediated signal transduction and expression, and decreased the activity of insulin degrading enzyme (IDE). IDE substrates include both insulin and AB, and indeed, these mice show increased AB1-40 and AB1-42 generation in the brain, corresponding to increased y-secretase and decreased IDE activity (Ho et al., 2004). Homozygous deletion of IDE in mice results in hyperinsulinemia, glucose intolerance, and increased cerebral AB accumulation (Farris et al., 2003).

Diabetes and increased fasting glucose are also associated with a greater risk of vascular disease mediated by advanced glycation products (AGEs) and the receptor for advanced glycation end products (RAGE) (Emerging Risk Factors Collaboration et al., 2010, 2014). AB is a substrate for RAGE, and increased RAGE expression in the vasculature can facilitate transport of AB across the blood–brain barrier (BBB). Accordingly, inhibition of RAGE in mice prevents accumulation of AB in brain parenchyma (Deane et al., 2003).

Insulin resistance may lead to AD via pathologic mechanisms. Chronic hyperglycemia reduces expression of the BBB glucose transporter GLUT1, further contributing to a hypometabolic and a high stress central environment (Pardridge et al., 1990). Neuronal insulin resistance impairs neuronal glucose uptake and metabolism (Willette et al., 2015), which results in both direct neuronal dysfunction and increased generation of amyloid through upregulation of the amyloid precursor protein (APP) (Blass et al., 2000). Additionally, *in vivo* studies in mice have shown that impairment of glucose metabolism leads to hyperphosphorylation of tau characteristic of AD, suggesting that abnormal glucose metabolism may also contribute to neurofibrillary tangle formation (Planel et al., 2004). Insulin resistance increases the activity of glycogen synthase kinase-3 (GSK-3), which phosphorylates tau and leads to the formation of neurofibrillary tangles. It has been postulated that neuronal insulin resistance or “type 3 diabetes” may be a driver of AD (de la Monte and Wands, 2008), however, more evidence is needed to support this mechanism.

Human and rodent studies suggest that correction of insulin signaling modifies the course of AD. In humans with mild AD, intranasal insulin administration transiently improves cognition and levels of the pathogenic Aβ42 peptide (Craft et al., 2012). A high-fat, low-carbohydrate ketogenic diet, which is known to limit insulin production, in mice that genetically over-express Aβ resulted in a 25% reduction of amyloid burden compared to mice on a standard diet (Van der Auwera et al., 2005), and implementation of a low-carbohydrate diet in patients with MCI improved memory (Krikorian et al., 2012). Because insulin resistance involves both amyloid-dependent and amyloid-independent pathways, it is a risk factor that is insufficiently targeted by amyloid-focused interventions (Kaplan et al., 1991; Dechant et al., 1993).

#### Inflammation

Healthy neuronal function depends on the carefully balanced promotion of neurotoxin and cellular waste clearance and inhibition of inflammatory damage to neurons. The brains of AD patients feature prominent upregulation of cyclooxygenase (COX), cytokines, and complement (Heneka et al., 2010). Clinical studies reveal elevated serum levels of inflammatory cytokines [particularly TNF-a (Álvarez et al., 2007) and IL-6 (Hüll et al., 1996)] in patients with AD and reduction of AD risk with long-term use of COX-inhibiting non-steroidal anti-inflammatory drugs (NSAIDs) (In t’ Veld et al., 2001).

Animal models suggest that microglia, complement, and COX have both deleterious and protective effects in AD. Microglial activation resulting from neuronal cytokine burden and direct contact with Aβ causes production of neurotoxic substances (e.g., nitric oxide) leading to both neuronal apoptosis and clearance of amyloid and other neurotoxic products (Tuppo and Arias, 2005). Microglia may be protective due in part to the secretion of metalloproteinase, which degrades Aβ (Qiu et al., 1997; El Khoury et al., 2007). Transforming growth factor beta (TGF-β) is involved in neuronal development and synaptic plasticity, and impairment in TGF-β causes microglial activation, neuronal apoptosis, and increased Aβ degradation (Wyss-Coray et al., 2001). Complement activation exacerbates neuronal degeneration through C1q-mediated activation of the membrane attack complex (MAC) (Fonseca et al., 2004), which binds to neuronal membranes to cause cellular death. Finally, over-expression of COX doubles Aβ plaque formation (Xiang et al., 2002), while reduction of prostaglandin signaling through the EP2 receptor is associated with reduced beta-secretase 1 (BACE) cleavage of APP into Aβ (Liang et al., 2005). Ideally, interventions for AD prevention will reduce neurotoxic inflammation and amyloid genesis while preserving microglia- and cytokine-mediated neuronal protection.

#### Oxidative Stress

Oxidative stress is another primary pathway of cellular damage throughout the body. The toxic molecules are reactive oxygen species (ROS), which include superoxide anion, hydrogen peroxide, and hydroxyl radicals among many others. These ROS have incomplete electron orbitals that can react with nucleic acids, protein, and lipids to cause dysfunction of many cellular components (Bayr, 2005). Brains of patients with AD demonstrate multiple stigmata of oxidative stress: increased lipid peroxidation, protein and DNA oxidation, and evidence of impaired mitochondrial function (Markesbery, 2005).

As one of the final pathways to cell death, oxidative stress is driven both by amyloid-dependent (Yatin et al., 1999) and amyloid-independent mechanisms (Ansari and Scheff, 2010). While cerebral amyloid can increase ROS through induction of an inflammatory response (Johnstone et al., 1999), there are multiple other pathways of ROS generation that likely contribute to the neuronal damage in AD. The brain consumes about 20% of the body’s oxygen, and the high activity of the electron transport chain (ETC) in neural mitochondria generates a large amount of hydrogen peroxide and other ROS (Halliwell, 1992). Neurotransmitter synthesis enzymes such as monoamine oxidase and tyrosine hydroxylase produce ROS during normal catecholamine synthesis. Finally, glutamate, the major excitatory neurotransmitter, increases the production of arachidonic acid and its ROS byproducts through NMDA receptor activation (Dumuis et al., 1988). Hypoglycemia can also lead to ROS formation due to disruption of the ETC. Disruption of the ETC will lead to decreased ATP production and cellular energy deprivation, eventually leading to apoptosis. ROS production will also affect other key enzymes responsible for creating compounds that scavenge free radicals [e.g., the alpha-ketoglutarate dehydrogenase complex (a-KGDHC) of the TCA cycle]. Without optimal function of this thiamine-dependent enzyme, which may result from thiamine deficiency or ROS overproduction, there is an increased susceptibility to cellular damage by ROS (Park et al., 1999; Gibson and Shi, 2010). By blocking the production of ROS byproducts, this offers a possible mechanism explaining the protective effects seen with NSAID use (Ghosh et al., 2017). Given the pleotropic effects on cellular function, reduction of ROS has been identified as a modality for the prevention of multiple forms of neurodegenerative disease (Coyle and Puttfarcken, 1993).

### Neurologic Mechanisms of Alzheimer’s Disease Pathogenesis: Trophic Factors, Amyloid Burden, and Calcium Toxicity

#### Trophic Factors

The severity of the AD clinical phenotype can be thought of as a balance between neuronal degeneration from amyloid, oxidative stress, and other neural compensatory mechanisms like plasticity and cognitive reserve. Neural trophic factors, particularly nerve growth factor (NGF) and brain-derived neurotrophic factor (BDNF), are major mediators of compensatory plasticity and likely play a protective role in the development of AD (Kaplan et al., 1991). NGF and BDNF signal through the tyrosine kinase receptors TrkA and TrkB, respectively, to promote synaptic plasticity and prevent activation of the caspase system ensuring cell survival (Dechant et al., 1993). NGF’s anatomic specificity involves the survival of cholinergic neurons in the nucleus basalis (Cuello et al., 2010), damage to which plays an important role in producing the cognitive impairments of the clinical syndrome of AD. BDNF promotes plasticity and survival throughout the brain, but is particularly important for memory consolidation in the hippocampus (Egan et al., 2003). The brains of patients with AD reveal reduced mRNA levels of trophic factors (Phillips et al., 1991), a decline in serum levels of neurotrophic factors concomitant with progression of disease (Laske et al., 2006), and a reduction in trophic factor levels which rapidly leads to apoptosis in the presence of amyloid pathology (Hefti and Weiner, 1986; Guo et al., 1997).

#### Amyloid Burden

The generation of the Aβ peptide has long been thought to be one of the fundamental causes of AD. APP can be processed by alpha secretase and gamma secretase into the amyloid precursor protein intracellular domain (AICD) and protein P3, which are non-amyloidogenic and do not cause disease (O’Brien and Wong, 2011). Production of Aβ begins with the cleavage of APP by BACE into the soluble APP-β protein and a transmembrane fragment. The transmembrane fragment is then cleaved by gamma secretase into AICD and the Aβ peptide. Amyloid itself is thought to have pleiotropic downstream effects, from early synaptic dysfunction to microglial activation and inflammatory damage to oxidative injury (Hardy and Selkoe, 2002), as discussed above; however, many of these pathways can lead to cellular injury independent of amyloid and can cause amyloid deposition themselves (Lee et al., 2004).

### Calcium Toxicity

Calcium is a well-known mediator of cell death that causes both necrosis through activation of calcium-sensitive proteases and apoptosis through release of mitochondrial cytochrome c and activation of the caspase system (Gogvadze et al., 2001). Loss of membrane integrity and excessive neuronal excitation promote massive calcium influx into neuronal processes (Choi, 1994). Hypoxia may increase cytoplasmic calcium and mitochondrial calcium concentrations, increasing activation of calcium-dependent enzymes that may lead to ROS production and subsequent damage (Joseph and Gibson, 2007).

Disruptions of calcium signaling may be both a cause and result of Aβ formation: calcium stabilizes Aβ oligomers (Isaacs et al., 2006) and Aβ in turn induces unregulated flux of calcium through the cellular membrane (either through existing channels or new pore formation) (Demuro et al., 2010). Calcium toxicity is thought to be one of the basic mechanisms of neuronal death in AD, particularly through the activation of the calcium-activated proteinase calpain, which is widely activated throughout the brains of AD patients (Saito et al., 1993). In addition, dysregulation of endoplasmic reticulum (ER) calcium release has been observed in mouse models of AD (Qi and Shuai, 2016). Some of the genes implicated in AD regulate ER calcium transport as well as amyloid production (Supnet and Bezprozvanny, 2010). Systemic alterations of calcium binding are also seen in AD, but their significance for neuronal dysfunction remains unclear (Peterson and Goldman, 1986).

## Interventions for Alzheimer’s Disease Risk Reduction

A comprehensive plan for AD risk reduction should be founded on three guiding principles. First, the plan should consider non-amyloid mechanisms as amyloid may not be the only pathogenic driver of AD. Second, the earlier the intervention, the more effective the risk reduction. Third, interventions are considered in the context of the individual patient’s existing conditions as one size does not necessarily fit all.

Non-amyloid mechanisms may contribute to AD through both amyloid-dependent and amyloid-independent pathways. Accordingly, the role of risk reduction would primarily be to increase the resistance to non-amyloid AD pathology, while also reducing amyloid burden itself.

Risk factors for AD such as reduced cerebral blood flow can precede clinical disease onset by years. Furthermore, AD pathological changes progress over time as the disease becomes more serious (Bancher et al., 1993) suggesting that the chance of therapeutic success is greater at earlier disease stages. The importance of early targeted treatment has been shown with respect to insulin. Intranasal insulin has been shown to improve cognition and/or memory in healthy persons (Benedict et al., 2004, 2007), obese men (Hallschmid et al., 2008), and additionally lower cerebrospinal fluid levels of AB42 in adults with amnestic MCI or mild to moderate AD (Reger et al., 2008; Craft et al., 2012).

Some interventions may only work in certain patients, and therefore a precision medicine approach is necessary. For example, the major pathologic driver in certain patients may be neuronal insulin resistance (de la Monte and Wands, 2008); the interventions targeting insulin resistance will be particularly effective in this group but less effective in patients whose primary driver is genetic alterations in amyloid production.

An important aspect of this approach is that not all modalities function for all individuals, even among those with similar risk factors and history. Management plans are crafted for an “average patient” based on prior evidence which incorporates a broad spectrum of data from heterogeneous patient cohorts that may be applicable to a particular patient (Schelke et al., 2016). Prior research addressed three etiologies (insulin resistance, dyslipidemia, and oxidative stress) underlying AD pathogenesis at the molecular level (Schelke et al., 2016). This review expands on the roles of these mechanisms in targeting modifiable risk factors to inform multi-modal, personally tailored interventions for potentially reducing AD risk.

### Systemic Interventions for Alzheimer’s Risk Reduction: Improving Glucose Metabolism and Insulin Sensitivity, Modulating Inflammation, and Reducing Oxidative Stress

#### Improving Glucose Metabolism and Insulin Sensitivity

Chronic high-carbohydrate and high-calorie diets can interfere with insulin signaling through the development of hyperinsulinemia and insulin resistance, leading to pancreatic β-cell dysfunction, and through changes in expression of adipose-derived adipokines, which interfere with target organ insulin signaling (Hotamisligil et al., 1993; Kim et al., 2001; Bastard et al., 2002; Howard and Flier, 2006). Conversely, diets that restrict high glycemic carbohydrates and calories can improve the adipokine profile (Ruth et al., 2013) and decrease insulin resistance (Parillo et al., 1992). Further, dietary ketones have been found to improve AD pathology and cognition in both animal models and clinical studies (Krikorian et al., 2012; Yin et al., 2016; Koppel and Swerdlow, 2017). Low-carbohydrate diets may thus both reduce the long-term neuropathology of AD and increase neuronal function (Schelke et al., 2016).

Evidence suggests that carbohydrate restriction, rather than caloric restriction itself, has the most potent effect on biomarkers of insulin resistance (Volek et al., 2004). However, patients with underlying insulin resistance or diabetes may benefit from a moderate-fat, low-carbohydrate Mediterranean diet more than a carbohydrate-restricted diet alone (Shai et al., 2008). Most human studies of AD have found that Mediterranean diets confer the lowest risk of AD, even in the absence of specific caloric restriction (Scarmeas et al., 2009). Thus, the success of the Mediterranean diet in AD may thus reflect underlying subclinical insulin resistance in many patients.

Other preventive interventions may also enhance glucose metabolism and insulin sensitivity. Exercise helps maintain euglycemia and reduces insulin resistance and the risk of AD (Watson and Craft, 2003) by increasing peripheral glucose sensitivity and glucose disposal, particularly at muscle. This is particularly true for resistance weight training, which increases the insulin sensitivity of fast-twitch muscle fibers and promotes more efficient glucose uptake (Ivy, 1997). Both sleep restriction (Ju et al., 2014) and hypercortisolemia are associated with AD, suggesting that sleep hygiene and stress management may reduce insulin resistance and the risk of AD by restoring a healthy baseline similar to the expected effects of dietary modification. Sleep restriction reduces systemic insulin sensitivity through increased production of nighttime growth hormone and cortisol (Knutson et al., 2007) and decreases cerebral glucose metabolism (Thomas et al., 2000). Melatonin is frequently used to treat sleep disturbance in AD and may be helpful for a risk reduction regimen (Cardinali et al., 2002). Stress with concomitant hypercortisolemia causes insulin resistance systemically (Andrews and Walker, 1999) and reduces glucose uptake and metabolism in hippocampal neurons (Virgin et al., 1991). Finally, there is evidence that lipoic acid can improve peripheral insulin sensitivity in type 2 diabetes through activation of the enzymes protein kinase B and phosphoinositide 3-kinase (Holmquist et al., 2007).

#### Modulating Inflammation

NSAIDs have been shown to reduce serum APP load (Skovronsky et al., 2001) and reduce cerebral amyloid burden (Lim et al., 2000) in animal models. In humans, prolonged NSAID use (at least 2 years) is associated with reduced risk of AD (Szekely et al., 2004). However, studies have found differing effects depending on the specific NSAID used (with ibuprofen showing the most consistent effects and selective COX inhibitors showing no effect) (Gasparini et al., 2004). In humans, a small study of perispinal administration of the TNF-a inhibitor etanercept demonstrated cognitive improvement in patients with AD (Tobinick, 2007), suggesting that modulation of cytokines and microglial activation may also modify the disease course.

Two non-pharmacologic methods also show promise in reducing risk of AD through modulation of inflammation. Diet has profound effects on the inflammatory response. High-carbohydrate, high-calorie meals, in particular, are associated with elevated C-reactive protein (CRP), IL-6 (Lopez-Garcia et al., 2004), and lipopolysaccharide (LPS, a potent activator of complement) (Ghanim et al., 2009) expression. The most potent dietary inducers of this systemic inflammatory response appear to be refined starches (e.g., white flour and its derivatives), refined sugar (e.g., sucrose and high-fructose corn syrup), artificial *trans*-fats, and saturated fats in the presence of high carbohydrate intake (Giugliano et al., 2006). CRP itself is directly neurotoxic and has been associated with AD (Duong et al., 1998), while the other nutritionally modulated inflammatory factors may contribute to AD pathology through the mechanisms outlined above or decrease overall cognitive function directly (Wärnberg et al., 2009).

In addition to diet, sleep and social engagement are known to modulate systemic inflammation. Sleep deprivation from 8 to 4 h significantly increases monocyte transcription of IL-6 and TNF-a (Irwin et al., 2006), both of which are associated with AD pathology (discussed above). Greater social engagement has been associated epidemiologically with reduced risk of AD (Saczynski et al., 2006) and biologically with a more robust natural killer cell immune response (Bower et al., 2003) and reduction in IL-6 (Friedman et al., 2007). While more data is needed to elucidate the relationships between nutrition, social engagement, inflammation, and AD, reduction of AD risk through modulation of inflammation will likely involve a combination of these lifestyle modifications and more targeted pharmacologic interventions like NSAID therapy.

#### Reducing Oxidative Stress

As one of the final pathways of neuronal damage in AD, oxidative stress may be essential to treat for risk reduction. This may be particularly important for certain patient subgroups; apoE4 (apolipoprotein E4) carriers, for example, have increased markers of oxidative stress and inflammation (Jofre-Monseny et al., 2008). This may be a primary reason for the increased AD risk with an apoE4 allele (Jofre-Monseny et al., 2008). Other systemic conditions like metabolic syndrome also increase baseline oxidative burden; particular attention to these interventions may be warranted in these groups as well (Jofre-Monseny et al., 2008).

Reduction of oxidative stress involves either inhibition of ROS production or acceleration of ROS clearance. Intake of carbohydrate-rich meals stimulate the production of ROS from mononuclear cells (Patel et al., 2007), while carbohydrate restriction increases the efficiency of electron flow through mitochondrial ETC and reduces ETC generation of ROS (Guarente, 2008). Hyperglycemia also increases ROS production and oxidation of the ApoE protein in astrocytes, preventing ApoE-mediated proteolysis of Aβ. Given the association of AD with mitochondrial oxidative damage (Hirai et al., 2001), carbohydrate control may reduce the risk of AD through reduction of oxidative stress along with its roles in insulin resistance and inflammation. In addition to diet, stress and cortisol also appear to induce ROS production causing direct neuronal damage and downregulation of matrix metalloproteinase-2 expression, preventing degradation of Aβ (Lee et al., 2009). The vitamins B6, B9 (folic acid), and B12 are involved in the metabolism of homocysteine, which is associated with AD and is a known stimulator of ROS production (Seshadri et al., 2002). These B vitamins are necessary to convert homocysteine to methionine/cysteine, thus, preventing homocysteine-stimulated ROS production. Methylated forms have been found to delay cortical and white matter atrophy, yet further study is needed to determine the optimal form, dose and patient population in the preventative spectrum (e.g., based on nutrigenomics such as MTHFR mutations) for which B-vitamin supplementation is best suited (Shankle et al., 2016).

Other interventions may be able to improve clearance of ROS through free-radical scavenging. Multiple animal studies suggest that exercise increases the activity of antioxidant enzymes (AOEs) such as catalase and superoxide dismutase (Somani and Husain, 1996) and reduces the products of neuronal lipid peroxidation (Devi and Kiran, 2004). As exercise prevents declines in hippocampal function in humans (Intlekofer and Cotman, 2013) and decreases amyloid burden in rodents (Adlard et al., 2005), an active exercise regimen may reduce the risk of AD through reduction in cerebral oxidative stress. While aerobic exercise preferentially improves insulin sensitivity, the regularity of exercise is central to reduction of oxidative burden and risk of AD. Short episodes of exercise without a regular regimen increase oxidative burden (Radak et al., 2008) while regular exercise (at least three sessions per week) both decreases oxidative burden and reduces risk of AD (Larson et al., 2006).

Finally, certain dietary supplements may reduce ROS. Consumption of antioxidant omega-3 polyunsaturated fatty acids (PUFAs) is epidemiologically associated with reduced risk of AD (Morris et al., 2003), and n-3 PUFAs reduce amyloid burden (Lim et al., 2005) and oxidation-induced loss of postsynaptic proteins in AD models (Calon et al., 2004).

### Neurologic Interventions for Alzheimer’s Disease Risk Reduction: Addressing Trophic Factors, Reducing Amyloid Burden, and Managing Calcium Toxicity

#### Addressing Trophic Factors

While some groups are studying gene delivery of NGF and BDNF as a possible therapeutic target for AD (Tuszynski and Nagahara, 2016), it is possible that increasing expression of these factors before patients are symptomatic may reduce risk or delay the clinical onset of AD. Both exercise and reduction of hypercortisolemia may promote neural plasticity through increased production of neurotrophic factors. Exercise, particularly regular resistance training for at least 5 weeks, has been shown to increase circulating serum levels of neurotrophins (especially BDNF) in humans (Yarrow et al., 2010) which may mediate the reduced risk of AD seen in individuals who exercise consistently (Radak et al., 2010). Molecularly, resistance exercise stimulates muscular release of FDNC5 and its cleaved product irisin, which can induce hippocampal expression of BDNF (Wrann et al., 2013) (the corresponding mechanism for NGF is unknown). More limited data suggest exercise can induce glial cell line-derived factor (GDNF) (McCullough et al., 2013) which promotes neuronal survival (Lin et al., 1993). Exercise increases expression of IGF-I, with some data indicating resistance exercise is more effective for women (de Souza Vale et al., 2009; Baker et al., 2010). In rats, exercise or IGF-I administration has been shown to increase hippocampal neurogenesis. However, treatment with IGF-I blocking serum prevents exercise-induced neurogenesis (Trejo et al., 2001). Exercise also stimulates expression of vascular endothelial growth factor (VEGF) in the murine hippocampus, while blocking VEGF reduces exercise-induced proliferation of stem cell and committed neuronal progenitors in the hippocampus (Fabel et al., 2003). Postmortem analyses of subjects with AD shows reduced capacity for IGF-I signaling (Steen et al., 2005).

Acute and chronic stress and concomitant hypercortisolemia reduce plasticity throughout the brain, particularly in the hippocampus (McEwen, 1999). This is partially mediated through reductions in hippocampal BDNF expression (Murakami et al., 2005). Lifestyle modifications to minimize stress and resulting hypercortisolemia—which include cognitive behavioral therapy, mindfulness training, and other approaches (Greenberg, 2011)—may thus promote plasticity and neuronal survival and modulate AD risk.

One potential preventive modality that has been linked to neurotrophic factor expression is hormone therapy (HT), though the data are mixed. In animal models, testosterone administration increased concentrations of NGF and its receptor in the hippocampus and forebrain (Tirassa et al., 1997). Similarly, estrogen and progesterone administration increased hippocampal levels of BDNF (Gibbs, 1998). In humans, some evidence indicates that early HT in women may be protective particularly in patients with two apoE4 alleles (Zandi et al., 2002). More specifically, transdermal 17β-estradiol with oral progesterone reduced Aβ deposition in postmenopausal women and was superior to oral conjugated equine estrogen with progesterone (Kantarci et al., 2016). In women older than 65 years, however, oral conjugated equine estrogen-progestin therapy may increase the risk of AD (Henderson, 2014). Further examination of the role of HT in AD prevention is certainly necessary, particularly for younger subjects.

#### Reducing Amyloid Burden

Multiple interventions may modulate amyloid burden directly, outside of systemic effects on insulin resistance, inflammation, and oxidative stress. The most targeted of these are the anti-amyloid antibodies like aducanumab and solanezumab, which hypothetically bind to amyloid and allow immune-mediated clearance of the protein. Aducanumab and solanezumab (AAIC 2017, AD/PD 2017) have successfully reduced amyloid burden in humans (Sevigny et al., 2016) and solanezumab is the trial agent in an AD prevention trial (the A4 study) (Sperling et al., 2014). Results from these ongoing and future trials will give us more information on the utility of these agents, especially considering the multiple clinical trial failures. Given the unclear role of amyloid in AD pathogenesis, targeting of amyloid must be a part of a broader preventative plan that addresses other etiologic mechanisms such as those discussed above.

Along with these targeted therapies, certain hormonal therapies and supplements may prevent Aβ accumulation. In human cell lines, estrogen acts through the extracellular regulated kinases (ERKs) to promote alpha secretase cleavage of APP into the non-amyloidogenic P3 protein and facilitates microglial clearance of Aβ (Pike et al., 2009). However, given the diverse regimens of HT used in clinical trials, it is difficult to assess its efficacy *in vivo*. In addition, the supplement curcumin (found naturally in turmeric) has been shown to reduce Aβ plaque development in mouse models possibly through reduced transcription of ApoE, which facilitates binding of Aβ into pathologic oligomers (Lim et al., 2001). Few human trials have been completed thus far, yet oral curcumin supplementation was not clinically effective for subjects already diagnosed with AD dementia. In this study, there was a lack of biochemical efficacy as well, as plasma levels were low (suggesting inadequate absorption). A recent double-blind, placebo-controlled trial found that twice-daily oral consumption of 90 mg of a bioavailable form of curcumin led to memory and attention benefits in non-demented adults over 60 years of age. Positron emission tomography (PET) imaging suggest the benefits are associated with decreased amyloid burden and tau accumulation (Small et al., 2017).

Finally, both the APP and BACE genes contain sequences for the binding of the glucocorticoid receptor, and administration of glucocorticoids to mice increases levels of APP, BACE, and Aβ (Green et al., 2006). While stress management may prevent development of pathology through many of the mechanisms outlined above, it may also directly reduce transcription of these proteins that are critical in the amyloid cascade.

#### Managing Calcium Toxicity

Two interventions have been shown to reduce calcium toxicity in animal models and may be useful for AD risk reduction. In a rat model of AD, Aβ-induced calcium flux through L-type calcium channels was partially ameliorated with administration of vitamin D (Dursun et al., 2011). Similarly, vitamin D was found to protect hippocampal neurons from excitotoxic insults through downregulation of calcium channels (Choi, 1994). Vitamin D deficiency is a known risk factor for AD and has been linked to disruptions in calcium signaling (Littlejohns et al., 2014). However, it is unclear whether supplementation in humans beyond correction of deficiency has any role in risk reduction.

In addition, sleep deprivation may disrupt calcium signaling in the hippocampus. In a rat model of aging, sleep deprivation caused increased hippocampal calcium influx in response to excitation compared to well-rested control animals (de Souza et al., 2012). It is too soon to tell if dysregulation of calcium signaling underlies the profound cognitive impairments seen with sleep deprivation.

## A Multifactorial Program to Reduce Ad Risk

**FIGURE 2:**
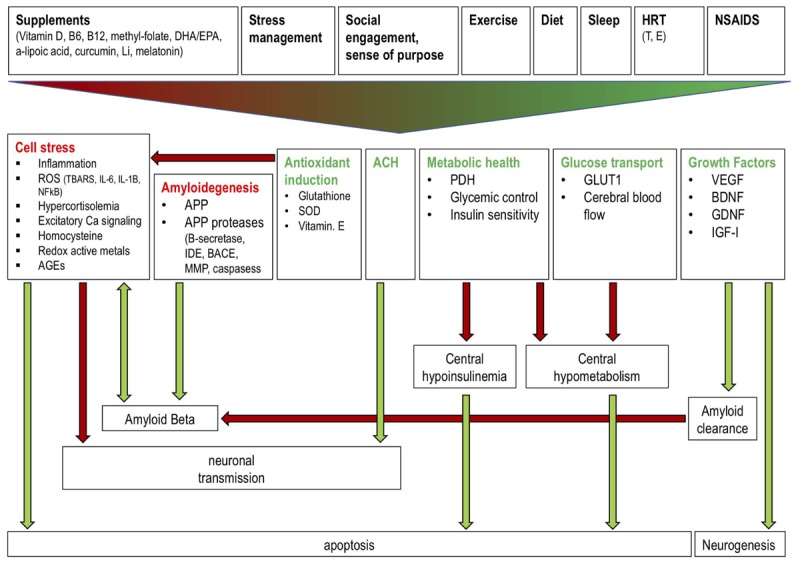
Effects of a polytherapeutic approach on AD mechanisms. Interventions (supplements, stress management, social engagement and sense of purpose, exercise, diet, sleep, HRT, NSAIDs) collectively reduce (red text) and stimulate (green text) factors which influence amyloid beta generation and clearance, central insulin and glucose metabolism, neuronal transmission, and neurogenesis. Green and red arrows indicate one factor increasing or decreasing another, respectively. Antioxidant induction reduces cell stress thereby preventing reduced neuronal transmission and enhanced AB generation and apoptosis. Amyloidogenic factors increase AB production while growth factor (e.g., IGF) mediated amyloid clearance reduces AB. ACH improves neuronal transmission. Metabolic health and glucose transport factors prevent a central hypoinsulinemic and hypometabolic state which prevents apoptosis. Growth factors stimulate neurogenesis. Li: lithium; DHA: docosahexaenoic acid; EPA: eicosapentaenoic acid; HRT: hormone replacement therapy; NSAIDS: non-steroidal anti-inflammatory drugs; T: testosterone; E: estrogen; ROS: reactive oxygen species; TBARS: Thiobarbituric acid reactive substances; IL: interleukin; AGEs: advanced glycation end products; IDE: insulin degrading enzyme; SOD: superoxide dismutase; PDH: pyruvate dehydrogenase; GLUT1: glucose transporter 1; VEGF: vascular endothelial growth factor; BDNF: brain derived neurotrophic factor; GDNF: glial cell derived neurotrophic factor; IGF: insulin-like growth factor.

The diversity of mechanisms and interventions described above offer multiple opportunities for clinicians to begin incorporating AD risk reduction into clinical practice (**Figure 2**). Most risk-reducing interventions, similarly to those for cardiovascular disease (CVD), involve the restoration of a “healthy baseline.” Along these lines, a successful dietary intervention doesn’t necessarily improve serum lipids and blood pressure; rather it corrects them to levels we would find in a healthy individual at low risk for CHD and AD. In this context, effective interventions involve modifying suboptimal components of health to levels that are less deleterious. For AD, this includes the four interventions directed at systemic health: diet, exercise, sleep, and stress management. As described above, dietary patterns that appear most efficacious include a carbohydrate-restricted diets, with Mediterranean diets being particularly useful for older individuals or those with existing insulin resistance. Many of the benefits from exercise are likely mediated through resistance training mediated alterations in neurotrophin levels and muscle insulin sensitivity, though most epidemiologic studies suggest that the regularity of exercise (at least three times per week) is also critical. It is likely that a mixture of aerobic and anaerobic elements is reasonable. Studies of sleep deprivation show worsening of biomarkers and function below 7–8 h of sleep (beginning around 11 PM), though the effects on AD risk are difficult to assess given that sleep is abnormal early in AD progression (Prinz et al., 1992). Finally, reduction of hypercortisolemia through stress management is the least well-specified of these domains, but presumably mindfulness and cognitive-behavioral exercises can also be beneficial.

Targeted interventions that go beyond maintenance of systemic health, including NSAIDs for COX-mediated inflammation, B vitamins and PUFA supplementation for reduction of oxidative stress, and HT for reduction of amyloid burden and increased neurotropic factors, are not supported by definitive evidence to recommend a “one size fits all” prescription solely for AD risk reduction. In patients with comorbidities for whom these therapies would be useful, however, AD risk reduction is an additional reason to favor prescription. Patient selection is critical, though, and many of these therapies are likely more useful in narrowly defined populations [e.g., B vitamin supplementation for those with elevated homocysteine or preexisting deficiencies, and HT for women early in perimenopause for a restricted period of time (Seifan and Isaacson, 2015)].

## Conclusion

Six primary systemic and neurologic mechanisms have been described—dysregulation of glucose metabolism, inflammation, oxidative stress, trophic factor release, amyloid burden, and calcium toxicity—and are associated with AD risk, making them likely to play important roles in the pathogenesis of AD. While a full review of potential mechanisms, including pleotropic effects of some interventions (e.g., statins, flavanols, intermittent fasting, and cognitive engagement) is beyond the scope of this review, management that target these mechanisms may substantially reduce risk for late-life AD and/or delay or possibly prevent the disease. In the clinic, these components of the systemic milieu should be the focus of AD prevention with addition of more targeted therapies for specific risk factors. While we await the results of ongoing trials for anti-amyloid preventive agents, the pathogenesis of AD likely extends far beyond amyloid. A multimodal approach targeting the complex and related mechanisms described above will be crucial for risk reduction.

From a practical clinical perspective, this integrated, evidence-based, intervention strategy based on translational science and evidence from observational studies simultaneously targets multiple modifiable risk factors, potentially benefiting brain and body health (Smith and Yaffe, 2014; Norton et al., 2014; Galvin, 2017). Past studies have used population-attributable risk models to estimate that one in three people with AD may have been able to avoid the disease based on a host of interventions (Norton et al., 2014). There is ample time for individuals at-risk to make a number of lifestyle changes toward both primary and secondary (pre-symptomatic) AD prevention. In fact, one study projected the effect of such risk factor reduction on AD prevalence; it found that even a modest reduction (10–25%) in seven risk factors (T2D, midlife hypertension, midlife obesity, smoking, depression, cognitive inactivity or low educational attainment, and physical inactivity) could potentially prevent as many as 1.1–3.0 million AD cases worldwide (Barnes and Yaffe, 2011).

While we expect that maintenance of a healthy systemic milieu will produce dividends in AD risk reduction and early intervention, the best approach may be more nuanced and would modify the prevention regimen based on the individual’s genetics, biomarkers, and lifestyle (Schelke et al., 2016). The definitive clinical precision medicine approach to AD risk reduction remains to be formalized, but the foundations for intervention likely involve addressing the mechanisms described in this review. From a practical clinical perspective, this approach would be applicable to the tens of millions of patients worldwide at risk for or already experiencing the earliest pre-symptomatic (Stage 1) and mildly symptomatic (Stage 2) pre-dementia phases of AD. Ultimately, AD may represent an end stage of multiple pathologic processes; identifying and targeting these processes simultaneously will allow for tangible results in the effort to reduce risk and improve patient outcomes.

## Author Contributions

MS, PA, DP, CG, RKa, RKr, LM, RI, BK, MM, OS, and AR contributed to manuscript writing and the literature review. MS, PA, and RI contributed to the conception and design of the work and the final approval of the version to be published.

## Conflict of Interest Statement

RI has served as a consultant for Lilly, Neurotrack, and 23 and Me. The other authors declare that the research was conducted in the absence of any commercial or financial relationships that could be construed as a potential conflict of interest.
